# GWAS3D: detecting human regulatory variants by integrative analysis of genome-wide associations, chromosome interactions and histone modifications

**DOI:** 10.1093/nar/gkt456

**Published:** 2013-05-30

**Authors:** Mulin Jun Li, Lily Yan Wang, Zhengyuan Xia, Pak Chung Sham, Junwen Wang

**Affiliations:** ^1^Department of Biochemistry, LKS Faculty of Medicine, The University of Hong Kong, Hong Kong SAR, China, ^2^Shenzhen Institute of Research and Innovation, The University of Hong Kong, Shenzhen, Guangdong 518057, China, ^3^Department of Anaesthesiology, LKS Faculty of Medicine, The University of Hong Kong, Hong Kong SAR, China, ^4^Centre for Genomic Sciences, LKS Faculty of Medicine, The University of Hong Kong, Hong Kong SAR, China, ^5^Department of Psychiatry LKS Faculty of Medicine, The University of Hong Kong, Hong Kong SAR, China and ^6^State Key Laboratory in Cognitive and Brain Sciences, The University of Hong Kong, Hong Kong SAR, China

## Abstract

Interpreting the genetic variants located in the regulatory regions, such as enhancers and promoters, is an indispensable step to understand molecular mechanism of complex traits. Recent studies show that genetic variants detected by genome-wide association study (GWAS) are significantly enriched in the regulatory regions. Therefore, detecting, annotating and prioritizing of genetic variants affecting gene regulation are critical to our understanding of genotype–phenotype relationships. Here, we developed a web server GWAS3D to systematically analyze the genetic variants that could affect regulatory elements, by integrating annotations from cell type-specific chromatin states, epigenetic modifications, sequence motifs and cross-species conservation. The regulatory elements are inferred from the genome-wide chromosome interaction data, chromatin marks in 16 different cell types and 73 regulatory factors motifs from the Encyclopedia of DNA Element project. Furthermore, we used these function elements, as well as risk haplotype, binding affinity, conservation and *P*-values reported from the original GWAS to reprioritize the genetic variants. Using studies from low-density lipoprotein cholesterol, we demonstrated that our reprioritizing approach was effective and cell type specific. In conclusion, GWAS3D provides a comprehensive annotation and visualization tool to help users interpreting their results. The web server is freely available at http://jjwanglab.org/gwas3d.

## INTRODUCTION

Recent studies on human genetics, such as The International HapMap Project (1) and 1000 Genomes Project (2), have identified a large number of genetics variants in the human genome. Furthermore, genome-wide association studies (GWAS) (3) and exome sequencing (4,5) are extensively used to globally investigate the relationship between genetic variants and human diseases/traits. By looking at the genomic location of the associated variants detected in GWAS, a large portion (∼88%) of them fall outside of coding regions, which are harder to interpret than the protein-coding variants (6). Therefore, elucidating the molecular function of genetic variants locating in the non-coding regions is critical to our full understanding of genetic disorders.

However, there are many difficulties and computational challenges in achieving this goal (7). One of the major difficulties comes from the unclear role of non-coding genetic variants in the relevant processes underlying disease/trait association. These variants could affect many biological activities including transcription, splicing, post-transcriptional regulation, translation initiation/elongation and post-translational modification (8). Previously, conservation information was frequently used to prioritize the functional importance of non-coding genetic variation (9,10). At the transcription regulation level, mutations in the promoter regions may impact the recruitment of RNA polymerase and other regulators, especially the binding of transcriptional factors (TFs) to the promoter region to initiate gene transcription. Tools such as is-rSNP (11), sTRAP (12) and regSNPs (13) have been successfully developed to evaluate the binding affinity affected by genetic variation. However, although algorithms that solely used TF motifs are effective in finding regulatory elements in the immediate promoter regions but may inevitably introduce a large number of false positives in the distal promoter/enhancer regions where the searching space becomes substantially larger. More and more studies showed that mutations within the distal regulatory elements, such as enhancer, insulator and silencer, could also disrupt or change the binding of TFs, nucleosome positioning signals and chromatin states. Furthermore, the locally changed chromosome conformation can block or create looping interaction between distal elements and promoter regions (14) and subsequently influence gene regulation. Unfortunately, few tools or resources have used such information to study genetic variants.

The Encyclopedia of DNA Elements (ENCODE) project has identified a comprehensive map of functional elements and active chromatin marks by advanced techniques such as ChIP-seq, DNase-seq, bisulfate sequencing, chromosome conformation capture and so forth. (15,16). Recent studies showed that disease-associated single-nucleotide polymorphisms (SNPs) detected by GWAS are significantly enriched in the regions that harbor functional elements, such as transcriptional factor binding sites (TFBSs), histone modification marked regions, DNase I hypersensitive sites (DHSs) and expression quantitative trait loci (16–19). Two recently published databases, HaploReg (20) and RegulomeDB (21), have used these regulatory signals and marks to annotate the genetic variants, which offer comprehensive resources on regulatory variation. On the other hand, different functional elements have been reported to function in a tissue/cell type-specific manner. SNPs associated with the same trait are likely to locate in active chromatin marks in the same/relevant cell type (22), implying the possibility of detecting regulatory signals using the chromatin marks of phenotypically relevant cell type. Computational algorithms including ChromHMM (23) and Seaway (24) have been successfully applied to scan different functional elements in the genome. Therefore, combinatory analysis of GWAS data and functional elements in a specific cell type to capture regulatory variants for a particular disease/trait are needed.

Here, we develop a web server GWAS3D (http://jjwanglab.org/gwas3d) to systematically analyze the probability of genetic variants affecting regulatory pathways and underlying disease/trait associations by integrating chromatin state, functional genomics, sequence motifs and cross-species conservation for a set of GWAS data or variant list. We first collected and curated genome-wide chromosome interaction (5C, Hi-C, ChIA-PET) data, enhancer/insulator/promoter marks [H3K4me1, H3K27ac, p300, CCCTC-binding factor (CTCF), DHS] and ChromHMM predicted functional elements in 16 different cell types. Using those regulatory regions, we mapped genetic variants to the reference genome and evaluated the binding affinity changes of regulatory factors by scanning 73 ENCODE motifs. Finally, we combined original GWAS signal, risk haplotype, binding affinity significance and conservation information to prioritize the genetic variants. In addition, the system provides comprehensive annotation and visualization to help users to interpret the results. Comparing with existing software and databases, GWAS3D uses the latest information to build a one-stop web-based tool for clinicians and biologists to evaluate the deleteriousness of disease/trait-associated variants that affect transcription regulation on a broader spectrum, especially on non-coding genetic variation.

## METHOD AND PIPELINE

### Data collection and processing

GWAS3D integrates multiple genome-wide experimental data to connect genetic variants with underlying gene regulation mechanism through high-dimensional regulatory interactions. We first collected and curated the experimental results of long-range interactions, for 16 different cell types, measured by high-throughput chromosome conformation capture technologies (5C, ChIA-PET and Hi-C) from the ENCODE project, Gene Expression Omnibus (GEO) database and published resources (Supplementary Table S1). We directly used 5C and ChIA-PET interactions in the database and processed the Hi-C interactions by the iterative correction and eigenvector decomposition (ICE) algorithm (25), which can largely reduce the false positives and biases. Some chromatin marks have been reported and validated as the active signals of enhancers, including histone modifications of H3K4me1 and H3K27ac, DHSs and E1A-binding protein p300 (26,27), we therefore extracted the related ChIP-Seq peaks for the above 16 cell types from ENCODE. We also collected ChIP-Seq data of CTCF-binding sites, which imply transcription repression and chromatin insulation. For predicted elements, we downloaded the ChromHMM genome-wide maps of chromatin state annotations for supported cell types and extracted the promoter, enhancer and insulator elements whose signals are predicted as ‘Strong’. We also merged the genomic profiles of three ENCODE tier 1 cell lines (GM12878, K562, H1 human embryonic stem cells) to support the ‘no cell type restriction’ option.

Genetic variants data sets were collected from dbSNP137 (28) and 1000 Genomes Project phase 1 release version 2 (29), which comprise 52 054 804 and 26 152 995 SNPs and Indels, respectively. We assigned reference allele and all alternative alleles to dbSNP137 variants and used biallelic variants for 1000 Genomes Project. The allele information was used to calculate the binding affinity of TFs. Linkage disequilibrium (LD) data for 11 populations were retrieved from the merged data of HapMap phases I + II + III. LD data for four 1000 Genomes Project super populations were computed and retrieved from MACH (30). Genomic coordinate of each locus was converted to GRCh37 hg19 by UCSC liftover tool. Variants with dbSNP ID were mapped to dbSNP137 using dbSNP merge file. In addition, annotations for genes and other DNA elements were downloaded from the UCSC Genome Browser. Furthermore, GWAS3D used position frequency matrices of 73 transcription factors motifs grouped by family (ENCODE motifs) from ENCODE web site, which provides a comprehensive resource of 245 known motifs curated from TRANSFAC, Jaspar and protein-binding microarray experiments, and 293 novel motifs discovered by motif finding tools (including MEME, MDscan, Weeder, AlignACE) using large numbers of ChIP-Seq data. To consider the regions with evolutionary constraint, we also used the conservative elements by genomic evolutionary rate profiling (31) and used these signals to prioritize the suspected deleterious variants.

### Pipeline

**Figure 1.**
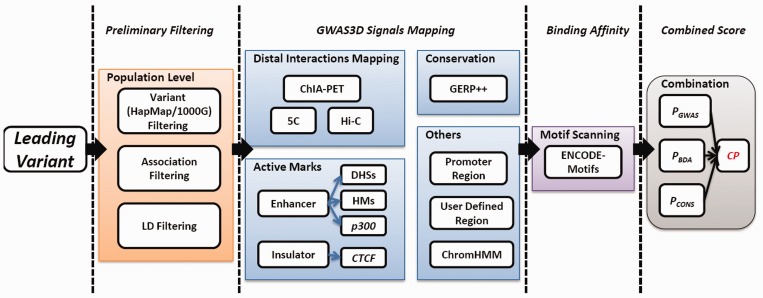
The workflow of GWAS3D (see the description of pipeline for details).

Given a set of GWAS data or a SNP list, GWAS3D can detect the variants’ regulatory effects such as the assigned population haplotype, the experimentally derived genetic/epigenetic signals, the predicted change of transcription factor binding affinity on different alleles and sequence conservation in a particular cell type. The server further calculates the combined effect of each variant and prioritizes them based on the probability of affecting gene regulation. The overall workflow of GWAS3D is shown in Figure 1.

#### Preliminary data filtering

The system accepts inputs either from an association study or a SNP list. Many formats are supported, including the Plink-like (32) format, VCF-like format, single dbSNP ID and variant chromosome position. The input of association *P*-value is compulsory when the GWAS effect size is considered for prioritization. A user-defined *P*-value cut off is applied to filter out the less significant SNPs and to reduce data volume. SNPs or Indels will be checked and mapped to dbSNP137 or 1000 Genomes Project variants. Variants not using VCF-like format will be assigned respective alleles according to dbSNP137 then 1000 Genomes Project. The web server will filter the variant not mapped onto either dbSNP137 or 1000 Genomes Project unless VCF-like format is used. Then, it would fetch all variants in LD of each aforementioned leading variants by user-defined LD standard (HapMap or 1000 Genomes Project), population and r-square (*r*^2^) cut off.

#### Identifying GWAS3D regulatory signals

Cell-type specific marks, including genome-wide long-range interactions, active promoter/enhancer/insulator marks, predicted ChromHMM maps, as well as a user-defined promoter region, are then used to identify the potential regulatory effects of the variants. We defined a variant mapped onto any of these regions as a ‘GWAS3D signal’, which implies a relevant regulatory function such as affecting distal interaction in high dimension or direct promoter activity of a target gene. The variants that are not mapped to any regulatory regions are filtered out at this stage.

#### Computing the binding affinity effect by ENCODE-motifs

To quantitatively measure the difference on the binding affinity caused by different alleles of candidate variants with GWAS3D signals and its significance, we used a comprehensive TF motif set to evaluate the possible reduced or enhanced binding. We first computed the position weight matrices (PWMs) from position frequency matrices of all ENCODE motifs by converting normalized frequency value to log-scale value using the method described previously (33,34). Given a variant (*V*) with GWAS3D signal, we took 30 bp of surrounding sequence and constructed the mutated sequences between the reference alleles (*A_r_*) and the alternative alleles (*A_a__1_*, … *A_an_*). For user-selected motifs of TFs, we scanned these sequences using PWMSCAN (35) and fetched *P-*values represent the significance of each putative TF-binding site. We set a PWMSCAN *P*-value threshold (1E-3) to reduce the number of false positive bindings. We then measured the score of binding affinity change by calculating the log-odds (LOD) of probabilities of paired binding sites for each motif (*m*):

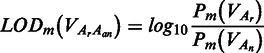

To estimate the statistical significance of binding affinity change, we performed permutations of on 52 054 804 SNPs in dbSNP137 and computed respective LOD of all ENCODE motifs to form the null distribution of binding affinity difference. We then used FastPval (36) to compute the *P*-value of each LOD from aformentioned empirical distribution.

#### Prioritization of regulatory variants

We combined the original GWAS signal, risk haplotype, binding affinity significance and conservation information to prioritize the leading variants (*L*) detected by GWAS. For each *L*, we first find all variants (*V*) with GWAS3D signal and in LD with *L* (*r*^2 ^> user selected cutoff). We then calculated *V*’s phenotypically associated effect (*P_GWAS_*) by dividing the GWAS *P*-value of *L* with the *r*^2^ between *V* and *L* in the user-specified populations. We then selected the most significant *P*-value of the LOD related to a specific TF motif (*P_BDA_*) to represent the binding affinity effect of the variant. We further mapped the variant to genomic evolutionary rate profiling++ (37) constrained elements and calculated the corresponding conservation *P*-value (*P_CONS_*). Using the *P*-values of aforementioned three independent measurements (GWAS, binding affinity, conservation), we performed Fisher’s combined probability test to calculate a combined *P*-value, *CP*, for each *V*. We then assigned the most significant *CP* from all the variants *V*s to the corresponding leading variant *L*. All the *L*s are then re-ranked according to their new *CP* values, with special focus on their regulatory effects.

#### Plotting the GWAS3D regulatory variants

To visualize global chromosome interactions among putative regulatory variants and their associated loci, GWAS3D also provide informative circle plots of high-dimensional chromosome interactions. We selected top significant variants (defined by the user) detected by GWAS3D and mapped them to RefGene for gene names or Cytoband for chromosome locations. We generated an intuitive circle graph using VIZ-GRAIL (38) with some modifications.

## WEB SERVER DESCRIPTION

### Usage and interface

The system accepts four formats for variants including either GWAS format such as Plink-like format and single dbSNP ID, or NGS format such as VCF-like format and single variant chromosome position resulting from high-throughput sequencing. LD information of different populations for both HapMap and 1000 Genomes Project is well supported by GWAS3D, which also allows users to define the cutoffs of association *P*-value and LD. More stringent settings will reduce the running time but some truely associated variants with moderate effect size might be lost. Also, GWAS3D provides information for 16 different cell types, which have been extensively investigated on chromatin states by recent ENCODE project, especially for long-range chromosome interactions. We recommend users to select the cell type that is relevant to the observed disease/trait in their GWAS/NGS study. Furthermore, GWAS3D allows users to choose relevant TF families and related known/novel motifs, which benefit capturing the binding affinity changes for a set of specific TFs. User can define a specific *P*-value cut off for putative TF-binding site scanning. Other settings, such as user-defined genomic regions and visualization options, including promoter definition, allowed number of variants and distal intentions for plotting, are also adjustable by the users.

**Figure 2.**
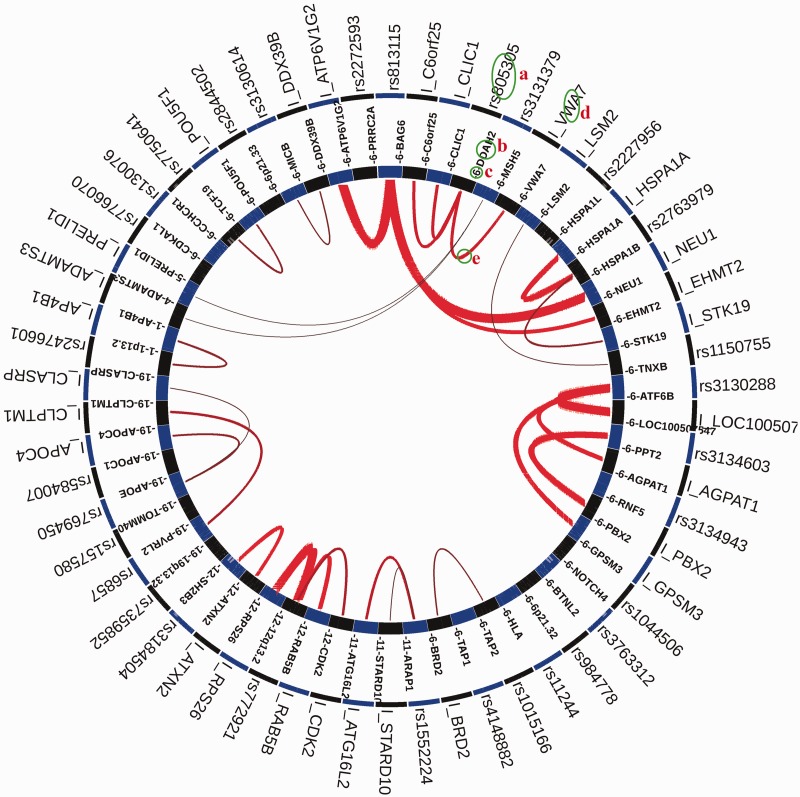
The circle plot of GWAS3D for all GWAS SNPs of diabetes mellitus from NHGRI GWAS Catalog based on the K562 cell line and CEU population. Significant GWAS3D results are presented by the circle plot. From the outer to inner, there are significant regulatory variants and distal interaction regions, genes and genomic loci, chromosome number and distal interaction indicators. For example, GWAS SNP rs805305 is detected as a significant regulatory variant by GWAS3D (**a**), this variant located on the intronic region of *DDAH2* (**b**) in chromosome 6 (**c**). One of the important regulatory features for this variant, which can be viewed from this plot, is that the region has a long-range interaction signal to another locus near *VWA7* (**d**), interactive elements with significant regulatory variant will start with ‘I_’). The red line indicated this signal (**e**), and the intensity of interaction is represented by width.

**Figure 3.**
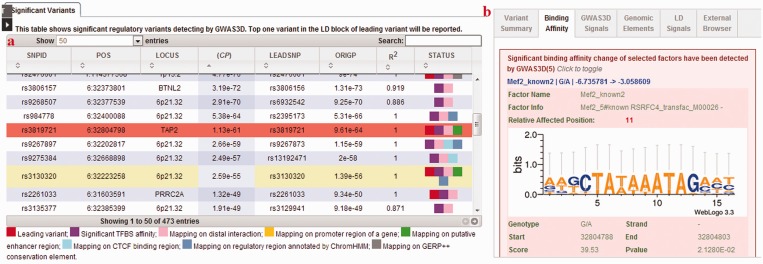
The ‘GWAS3D INFO’ page for detailed information of regulatory variant. The web page consists of two parts: (**a**) tabular viewer for significant variant detected by GWAS3D. (**b**) six annotation tabs of GWAS3D.

GWAS3D uses a series of user-friendly interfaces to display the results, which summarize the potential regulatory effects of these variants and facilitate the identification and selection of casual variants for follow-up experimental validation. The detected regulatory variants and their associated loci/interactions can be globally viewed from a circle plot in the left panel of ‘GWAS3D PLOT’ page (Figure 2). User can also check the overview of current GWAS3D run and download related information from right panel (Supplementary Figure S1). To query the detailed information of each variant with GWAS3D signals, we designed a variant prioritization table as well as comprehensive tab viewers in the ‘GWAS3D INFO’ page. In the prioritization table, the most significant regulatory variant in the LD of each leading variant is ranked by the significance of combined *P*-value. Also, variants with different type of GWAS3D signals are marked in different colors. For example, variants with significant TF-binding affinity change will be marked by purple stamp, and variants with active enhancer signal will be marked by green stamp. In the right tabs, user can identify the deleteriousness of selected variants by analyzing many annotations of its regulatory features in a dynamic manner (Figure 3).

### Annotation

For each variant detected by GWAS3D, we provided six annotations, including variant summary, binding affinity, GWAS3D signals, genomic elements, LD signals and external annotation. Users can systematically analyze the regulatory properties of variants based on these annotations. First tab is about variant summary, showing the important attributes related to the selected variant and reports the information of previous GWAS result recorded in GWASdb (39) for this variant. In the tab of binding affinity, we listed top five significant affinity differences of TF motifs with detailed binding sites information. For the GWAS3D signals information, we offered a tab to show all of mapped functional elements used in GWAS3D and related marks information. To help user identify all putative regulatory variants in the LD of observed leading variant, we used one additional tab to list related information. Lastly, three useful external browsers were encapsulated into the system to give broad annotations and predictions including GWASrap (40), RegulomeDB and UCSC ENCODE genome browser. User can directly fetch the information in an internal window.

### Evaluation

We first tested the web server using a well-studied locus known to be associated with plasma low-density lipoprotein cholesterol (LDL-C). We collected 17 associated SNPs in 1p13 region (Supplementary Table S2) genotyped in ∼20 000 individuals of European descent and ∼9000 African American individuals with LDL-C (41) and performed GWAS3D pipeline on those variants under HapMap CEU population and HepG2 cell type. We obtained five significant regulatory variants with distinguished GWAS3D signals and identified a leading variant rs12740374, as the top one in the prioritization table. The variant locates between genes *CELSR2* and *PSRC1*, and was scored a more significant *CP* (7.89E-46) than the original GWAS *P*-value (1.8E-42). GWAS3D reported that rs12740374 directly connected with two active enhancer marks (H3K27ac and DHS) and located in the conserved region. Importantly, binding affinity between allele G and risk allele T showed substantial difference for C/EBP transcription factors (Supplementary Figure S2). Those results were consistent with previous finding about the role of rs12740374 in the lipoprotein regulatory pathway (41). We also applied same associated SNPs set on YRI and CHB populations, besides the most significant leading SNP rs12740374, we further detected rs629301 that possibly disrupting the binding of transcription factor YY1, and other GWAS3D signals (Supplementary Figures S3 and S4). Another speculation is that this variant may influence the recognition and targeting of miR-199 in the 3′UTR of *CELSR2*. This variant was frequently reported as a highly associated signal in 1p13 region with LDL-C (42,43). Interestingly, we did not observe strong enhanced signals (active enhancer or promoter) at those GWAS-associated variants when we used non-liver cell type such as K562, H1-hESC and HeLa-S3. In contrast, CTCF-binding sites were observed around some of those associated variants, which may reflect the phenotypically cell type-specific association (Supplementary Figure S5) (22).

To further evaluate the ability of GWAS3D for detecting and prioritizing regulatory variants in a genome-wide manner, we collected 1370 associated SNPs with prostate cancer from NHGRI GWAS Catalog (6) and GWASdb (39). We detected 195 variants, which have GWAS3D regulatory signals (active promoter/enhancer/insulator marks, TFBS affinity changes, conserved elements), after applying the CEU population and RWPE1 cell type. Seven variants obtained more significant *CP* than the originals when analyzing top 20 putative regulatory variants in the prioritization table (Supplementary Table S3). Most of these significant variants exert the regulatory function of their associated loci by high linked LD variants other than leading SNPs. However, a noticeable result is a leading SNP rs6983267 that harbors many GWAS3D signals has been successfully validated by many functional studies for affecting enhancer activity (44,45).

We then quantitatively evaluated the performance of our method. We first collected 118 known regulatory variants from OregAnno database (46). We randomly selected three data sets from dbSNP with same number of genetic variants in each of the regulatory regions (promoter, intergenic and genome-wide). For each of aformentioned four SNVs list, we performed GWAS3D pipeline without considering GWAS *P*-value, cell type restriction and population LD. Wilcoxon rank-sum test showed significant differences between OregAnno and each random sets, with *P*-values of 0.0344, 0.0011 and 0.0052 for promoter, intergenic and genome-wide data set, respectively, whereas there are no differences among the three random data sets. The experiment demonstrated that GWAS3D pipeline gives higher scores to regulatory variants and thus differentiates them from random variants.

The SNVs detected by GWAS are associated with the disease/trait, but may not be the one with function implications. GWAS3D can find functional SNVs from GWAS SNVs through LD and other information. To assess this capability, we used GWAS3D pipeline to find functional SNV for each of the selected GWAS SNVs (118 top GWAS significant variants in intergenic and promoter regions from GWAS Catalog database). We found there were no significant differences between the GWAS SNVs and any of the three random data sets (all *P* > 0.05, Wilcoxon rank-sum test). However, the functional SNVs found by GWAS3D are significantly highly scored with *P*-values of 2.966E-05, 9.591E-08 and 3.034E-07 compared with the three random sets (Wilcoxon rank-sum test), respectively. Those results further confirmed the capability of GWAS3D in identifying functional regulatory variants (Supplementary Figure S6).

### Server design

We implemented the GWAS3D web server with a Perl-based web framework ‘Catalyst’, which provides a flexible programming interface on web development. Annotation information is stored in a back-end MySql database. We used Oracle Grid Engine as job management system for submitting tasks and offered three ways for users to retrieve their jobs: encrypted links, browser cookies and email notifications. jQuery and related UI components are used to construct dynamic web pages. GWAS3D is a one-stop framework with high usability and is freely available for academic use.

## DISCUSSION

We have designed a web-based tool to detect, prioritize and annotate the regulatory genetic variations in combination with experimental data and computational predictions. Particularly, this tool takes advantage of recently generated ENCODE data, especially the experimental long-range interactions as well as the active marks of functional elements, to predict variants in the putative TFBSs in distal and proximal promoter regions. GWAS3D is a tool dedicated to detect true functional variants that control gene regulation for genetic studies. Compared with recent software and databases such as VAAST, HaploReg and RegulomeDB, GWAS3D integrates more features and can be used in many scenarios. User can identify the most probable functional variant associated with interesting trait in one risk locus or prioritize the leading variants when given a full list of GWAS result or evaluate the deleteriousness of genetic variants affecting the gene regulation without any prior effect. GWAS3D also provides flexible configurations, such as human population, cell type specificity and TF family classification, for users to deal with different aspects of complex disease/trait. For example, user may select a matched cell type/tissue satisfying with a specific phenotype or manually define motifs of interested TFs used in following scanning when considering the tissue specificity of TFs. Recently, researchers found that the disease/trait-associated variants are highly related to active chromatin marks in relevant cell types (22). Therefore, these distinct features will greatly facilitate the discovery of regulatory variants under particular condition.

There are unbalanced genomic data of multiple domains for different cell types/tissues. For example, a lot of data were provided by ENCODE tier 1 cell lines (GM12878, K562, H1 human embryonic stem cells), whereas the data were few on tier 3 cell lines. It may potentially affect the quality of our annotation when applying GWAS3D pipeline to the cell lines having fewer data available. We therefore specially selected the 16 cell types, which included enough chromosomal looping data (5C or ChIA-PET or Hi-C) and important transcriptional markers data (H3K4me1, H3K27ac, DHSs, EP300 and CTCF). To cope with tissue/cell type limitation, we added a ‘without tissue/cell type restriction’ item in cell type selection option by merging the genomic profiles of three ENCODE tier 1 cell lines. Because the aforementioned three cell lines contain dynamic transcription signals from human normal adult cells, cancer cells and embryonic stem cells. In the future, we will continuously update the number of tissue/cell type when enough data are available for that cell line.

It was reported that many active chromatin marks are located in the intronic and exonic region of genes (47). Enhancers can also reside in intronic region of a gene to coordinate the looping with active promoter of another gene (48). Even for validated human fragments with enhancer activity (49), we found 30.42% of these fragments overlapped with coding region of genes. Thus, genetic variants not belonging to non-coding RNAs may also be associated with gene regulation. On the other hand, an exonic variant can associate with particular regulatory process by linking variants in the LD proxy. GWAS3D not only provides an efficient solution to interpret the regulatory role of genetic variation in the noncoding regions but also in other genic regions.

The computational process of our system is real-time, which is different from databases such as HaploReg and RegulomeDB, where the function annotations are pre-computed and stored in the database in advance. Therefore, it can dynamically deal with the genetic variants input by users with maximum flexibility. Despite large computational burden in the background when LD is considered, our system can finish the job of a meta GWAS data set (thousands of variants with moderate GWAS significance, *P* < 1.0 × 10^−5^) within a few hours even with LD from the 1000 Genomes Project. It will be much quicker when using HapMap LD. To exploit the regulatory properties of personal genomics data, GWAS3D accepts VCF-like format and can evaluate the deleteriousness of rare/novel variation altering gene regulation associated with personalized trait.

Furthermore, our system provides visualization and instant annotation for detected variants. Using the circle plotting, important regulatory variants and its affected regions, as well as the intra/interchromosomal interactions related to variants, can be intuitively displayed. Although many tools, such as SeattleSeq, ANNOVAR (50) and ENSEMBL VEP (51), can help users retrieve sufficient variant annotations, the integrative function annotation of GWAS3D will benefit users for instant query and broader range of information. Therefore, besides the genomic mapping information of variant (such as information of gene and other genomic elements), we offered several direct links to the servers of GWASrap (40), RegulomeDB and UCSC ENCODE genome browser in the internal windows of GWAS3D.

## SUPPLEMENTARY DATA

Supplementary Data are available at NAR Online: Supplementary Tables 1–3 and Supplementary Figures 1–6.

## FUNDING

Research Grants Council [781511M] of Hong Kong and National Science Foundation [91229105] of China. Funding for open access charge: Outstanding Young Researcher Award (OYRA) of The University of Hong Kong (2011–2012).

*Conflict of interest statement.* None declared.
